# An Unexpected Case of Intrapartum Pneumomediastinum

**DOI:** 10.1155/2019/4093768

**Published:** 2019-05-08

**Authors:** Drishti Madhok, Vinayak Smith, Erik Gunderson

**Affiliations:** ^1^Bundaberg Base Hospital, 271 Bourbong St Bundaberg, QLD 4670, Australia; ^2^Department of Obstetrics and Gynaecology, Monash University, 252 Clayton Road, 3168 VIC, Australia

## Abstract

The dyad of spontaneous pneumomediastinum and subcutaneous emphysema is collectively known as Hamman's syndrome. This rare complication is known to occur during the intrapartum period and its aetiology has been linked to the Valsalva maneuver in the second stage of labour. Nitrous oxide inhalation increases the risk. We present the case of a 21-year-old healthy woman who experienced these symptoms after nitrous oxide inhalation during the second stage of labour.

## 1. Introduction

Spontaneous pneumomediastinum is defined as free air in the mediastinum without any precipitating cause. Hamman was the first to describe the dyad of spontaneous pneumomediastinum in conjunction with subcutaneous emphysema in a labouring patient in 1939 [1]. Its incidence is now thought to be between 1 in 2000 and 1 in 100,000 deliveries [2]. Alveolar rupture leads to free air tracking through the mediastinum and then into the subcutaneous tissues [1]. We report a case of a 21-year-old healthy nulliparous female who had a normal vaginal birth followed by the presence of Hamman's syndrome 3.5 hours into her postpartum period.

## 2. Case Report

A 21-year-old healthy nulliparous female presented to a regional hospital in Australia in spontaneous labour at 39 weeks and 4 days of gestation. Her pregnancy was otherwise uncomplicated and she had no medical and surgical history of note.

The patient presented to the hospital with a cervical dilation of 4 cm. Her first stage of labour lasted 1 hr and 45 minutes and her second stage lasted 1 hr and 16 minutes, respectively. Analgesic treatment in the first stage included nitrogen oxide as required and 10 mg of intramuscular morphine. Analgesic used in the second stage was only nitrous oxide as required. An episiotomy was performed at delivery. She delivered a live born infant weighing 3690 g with APGAR scores of 9 and 10. The 3rd stage lasted 15 minutes with delivery of an intact placenta.

After delivery, she further requested nitrous oxide and had 10 mls of 1.0% lignocaine infiltrated into the perineum for an episiotomy repair.

Approximately 2 hours postpartum, the patient started complaining of sudden onset dyspnea and lower chest tightness. Her GCS was 15 at this time and her vital signs were oxygen saturation >95% on room air with a respiratory rate of 18 breaths per minute, pulse of 85 beats per minute, BP 130/78, and a temperature 37.2°C.

Physical examination at the time revealed swelling in the neck and jaw line and palpable crepitus in the anterior chest wall, neck, and jaw, consistent with subcutaneous emphysema.

Initial management included observation and pulse oximetry, as she was clinically stable and able to have a conversation without increase in chest pain or decrease in oxygen saturation.

**Figure 1:**
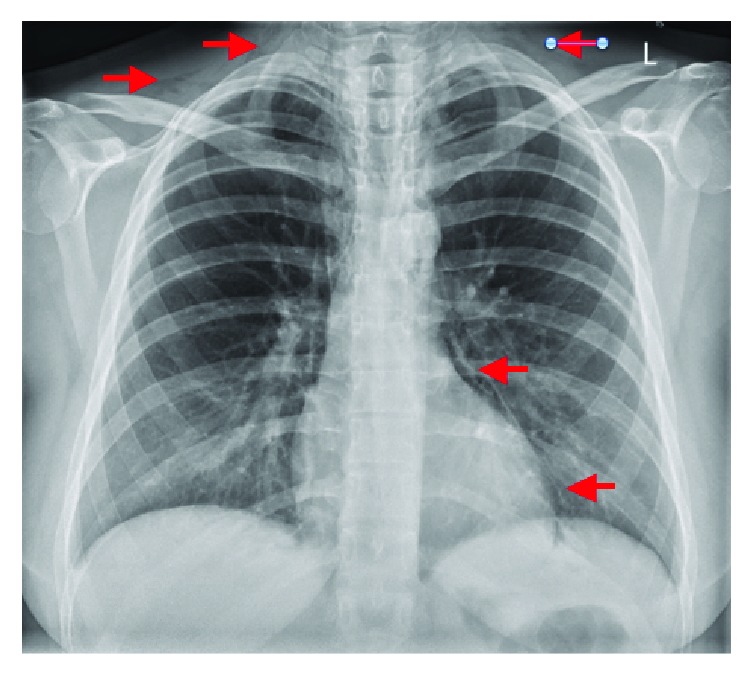
CXR confirmed the presence of free air in the pleural spaces and pneumomediastinum.

**Figure 2:**
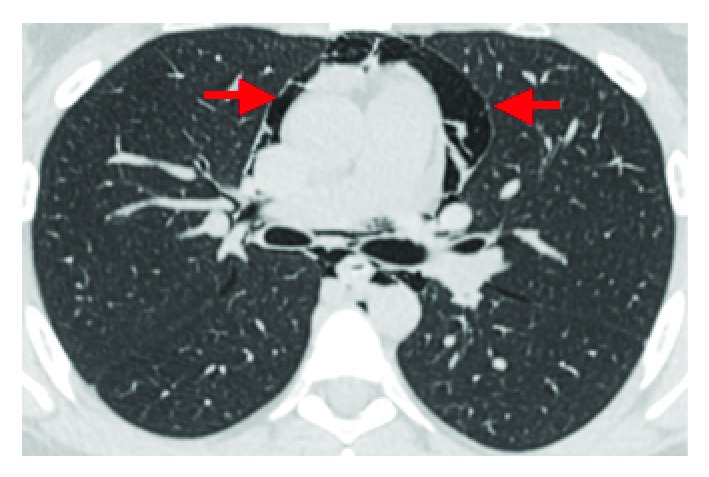
CT scan reconfirmed pneumomediastinum and subcutaneous emphysema and ruled out esophageal injury.

**Figure 3:**
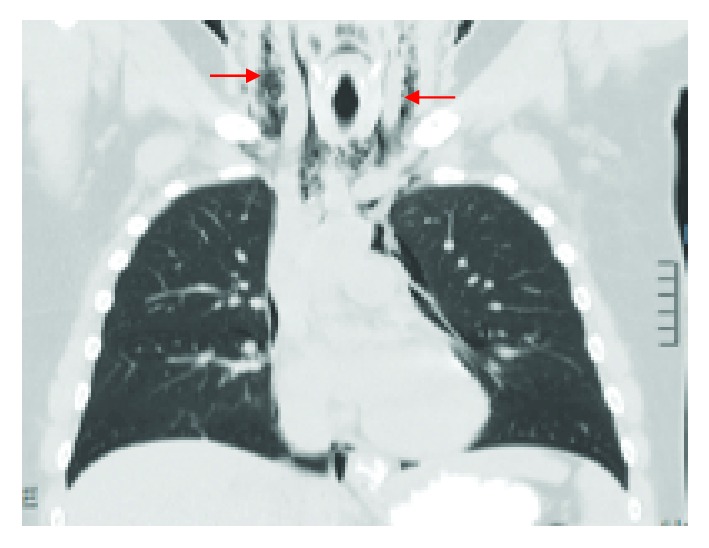
CT chest demonstrating extension of air in the cervical region.

Subsequently a chest X-ray (CXR) was obtained which demonstrated pneumomediastinum with air tracking superiorly into the pericardiac spaces (Figure 1). Following telephone consultation with a cardiothoracic team at our major tertiary referral center, a computed tomography (CT) contrast study was ordered as recommended. CT contrast reconfirmed pneumomediastinum and subcutaneous emphysema and ruled out esophageal injury (Figure 2). It also demonstrated extension of air in the cervical region (Figure 3).

The findings were once again discussed with the cardiothoracic team who advised expectant management due to the stability of the patient's clinical state.

**Figure 4:**
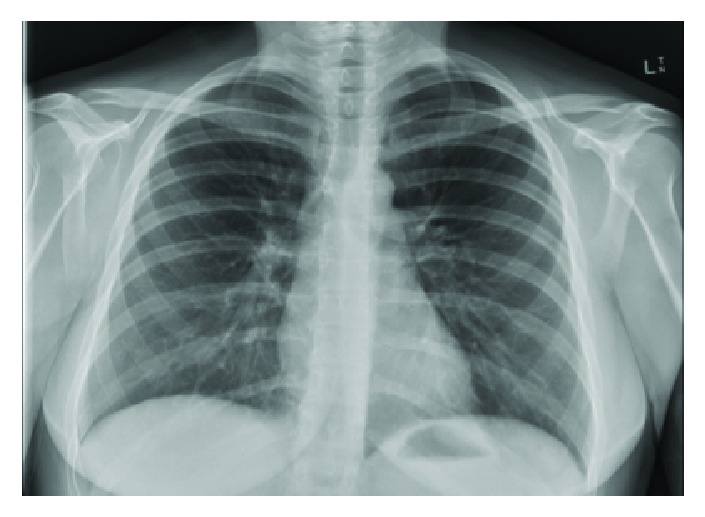
Normal CXR-resolution of free air in pleural spaces and pneumomediastinum.

Her postnatal course was uncomplicated thereafter and she only requested routine oral postpartum analgesia such as Panadol and Ibuprofen. Subcutaneous crepitus receded and became minimal by postpartum day 2 for the remainder of her hospital stay. She remained clinically stable and all observations were within normal limits. She was observed for 48 hours postpartum and was discharged on day 2 with the extended midwifery service. A repeat CXR done 2 weeks postpartum was normal and showed resolution of all findings (Figure 4).

## 3. Discussion

Hamman's syndrome is a rare complication of labour with a prevalence between 1:2000 and 1:100,000 deliveries [1]. Risk factors associated with it include nulliparous patients, prolonged second stage of labour, and any conditions that increase intrathoracic pressure such as coughing or heavy lifting [1]. There have been several documented cases [3–6] over the past 80 years.

### 3.1. Pathophysiology

The underlying pathophysiology of Hamman's syndrome is likely secondary to the Macklin effect [7]. In their experimental model, Macklin et al. proved that a rise in intra-alveolar pressure leads to terminal alveolar rupture which then allows alveolar air to pass through the perivascular interstitial tissue towards the hilum [7, 8]. The air remains trapped subsequently amongst the mediastinal structures but may eventually leak into the surrounding soft tissues [7, 9]. The air escape into the surrounding structures is facilitated through the fascial planes of the hilum, neck, and diaphragm around the aorta and oesophagus and pleural cavities [7, 8]. It is this air escape which causes the clinical signs of subcutaneous emphysema.

#### 3.1.1. Valsalva Maneuver

The Valsalva maneuver is thought to be contributory due to the increase in intrathoracic pressure it generates. There can be a repetitive and continuous rise in intrathoracic pressure with active pushing in the second stage of labour. There has been a demonstrable association between the length of Valsalva and risk of spontaneous pneumomediastinum [1, 3, 7, 10].

#### 3.1.2. Nitrous Oxide Utilization

Nitrous oxide utilization has been demonstrated as a causative mechanism of spontaneous pneumomediastinum during anaesthesia. It has a lower absorption into blood than the nitrogen component of air due to its lower blood: gas solubility index. This can potentiate abnormal extrapulmonary air spaces within the pulmonary parenchyma [11]. Therefore, contrary to the norm where air filled cavities decrease in size and eventually resolve, nitrogen oxide would tend to expand these cavities potentially leading to primary alveolar rupture or exacerbation of symptoms [12]. Once alveolar rupture occurs, continued use of nitrous oxide will lead to rapid gas dissection along the fascial tissue planes [12].

### 3.2. Clinical Presentation

#### 3.2.1. Symptoms

Commonly reported symptoms include [1, 5, 8]

(a) substernal pain/chest tightness due to distension of mediastinal space with air [1, 8]

(b) dysphagia due to the tracking of air into the laryngeal and pharyngeal tissue spaces [1, 8, 13]

(c) dysphonia due to the tracking of air into the laryngeal and pharyngeal tissue spaces [1, 1, 8]

(d) dyspnea due to increased air in the mediastinum which can result in pleural and diaphragmatic irritation [1, 8]

#### 3.2.2. Signs [1, 5, 8]

The signs include

(a) subcutaneous and retroperitoneal emphysema due to infiltration of air into the tissues [1, 8]

(b) obliteration of cardiac dullness or altered sounds over the heart due to distension of mediastinal space with air [1, 8]

(c) evidence of increased mediastinal pressure, cyanosis, and engorged veins: rising mediastinal pressure causing obstruction of blood flow to the heart (therefore, the peripheral veins become distended resulting in cyanosis and dyspnea) [1, 8]

(d) pneumothorax which is diagnosed clinically by shortness of breath and hypoxia and confirmed by CXR [8]

#### 3.2.3. Differential Diagnosis

When faced with a clinical presentation of Hamman's syndrome, the following differentials should be considered as well.

(a) Boerhaave's syndrome—spontaneous rupture of the esophagus—is precipitated by increased pressure and presenting with chest pain, dyspnea, pneumomediastinum, and emphysema. Unlike Hamman's syndrome, those who have Boerhaave's syndrome are not clinically stable and require immediate intervention [9, 10].

(b) Pericarditis: the sounds over the heart when air infiltrates the mediastinum consist of crackles/bubbles. They are accentuated in systole and when sitting up as opposed to lying down. This can mimic the “scratching” of pericarditis especially when in conjunction with sternal pain [1, 8].

(c) Myocardial infarction: when air tracks into the mediastinum the pain is usually beneath the sternum and can radiate over the chest and shoulders occasionally down the arms similar to angina [1, 8].

(d) Pulmonary embolus: when air tracks into the mediastinum it can also cause shortness of breath and chest pain also seen in pulmonary embolus [1, 4, 8, 9].

### 3.3. Management

Spontaneous pneumomediastinum is commonly a benign condition that can be treated conservatively [3–6, 9]. Bed rest, analgesia, and observation to prevent any other secondary complication are adequate [5]. Increasing inspired oxygen via nasal prongs or face mask will resolve any remaining alveolar distension. Symptomatic care can be managed similar to that of the bends from deep sea diving.

## 4. Conclusion

This case report documents the occurrence of Hamman's syndrome in an otherwise healthy 21-year-old patient. As a very rare complication of labour, we wish to add to the available literature. Commonly thought to occur as a result of alveolar barotrauma, this case highlights the interaction of Valsalva pushing and nitrous oxide use. Diagnosis is usually established via physical exam and routine CXR and/or CT. It is usually a benign condition that resolves spontaneously without significant morbidity.
